# Supplementary material for: Dynamic Alterations to Hepatic MicroRNA-29a in Response to Long-Term High-Fat Diet and EtOH Feeding
Source: Int J Mol Sci. 2023 Sep 26;24(19):14564. doi: 10.3390/ijms241914564 (PMC10572557; doi:10.3390/ijms241914564)
Supplement: Supplementary file 1 [file ijms-24-14564-s001.zip › ijms-2531299-supplementary.pdf]

Ingredient	High Fat Diet TD.06303	Calorie-Matched Diet TD.110196
	**g/Kg**	**g/Kg**
Casein	230	183
DL-Methionine	3.4	2.7
Sucrose, fine ground	213.7098	100
Corn Starch	80	431.2751
Maltodextrin	140	130
Vegetable Shortening, hydrogenated (Primex)	220	0
Soybean Oil	10	60
Cellulose	50	50
Mineral Mix, AIN-93G-MX (94046)	46	36.8
Calcium Phosphate, dibasic	3.3	2.72
Niacin	0.042	0.051
Calcium Pantothenate	0.0224	0.027
Pyridoxine HCl	0.0098	0.012
Thiamin HCl	0.0084	0
Thiamin (81%)	0	0.0105
Riboflavin	0.0084	0.0105
Folic Acid	0.0028	0.0033
Biotin	0.0003	0.0003
Vitamin B12 (0.1% in mannitol)	0.035	0.042
Vitamin E, DL-alpha tocopheryl acetate (500 IU/g)	0.1	0.15
Vitamin A Palmitate (500,000 IU/g)	0.0112	0.0033
Vitamin D3, cholecalciferol (500,000 IU/g)	0.0028	0.0033
Vitamin K1, phylloquinone	0.0011	0.0015
Choline Bitartrate	3.3	3.17
TBHQ, antioxidant	0.046	0.01
	**Percentage**	**Percentage**
Protein % by weight	20.4	16.2
Protein % kcal from	17.7	17.6
Carbohydrate % by weight	42.9	62
Carbohydrate % kcal from	37.1	67.3
Fat % by weight	23.2	6.2
Fat % kcal from	45.2	15.1
Kcal/g	4.6	3.7

Supplemental Table S1. Detailed information of high fat diet and calorie-matched diet.

Supplemental material

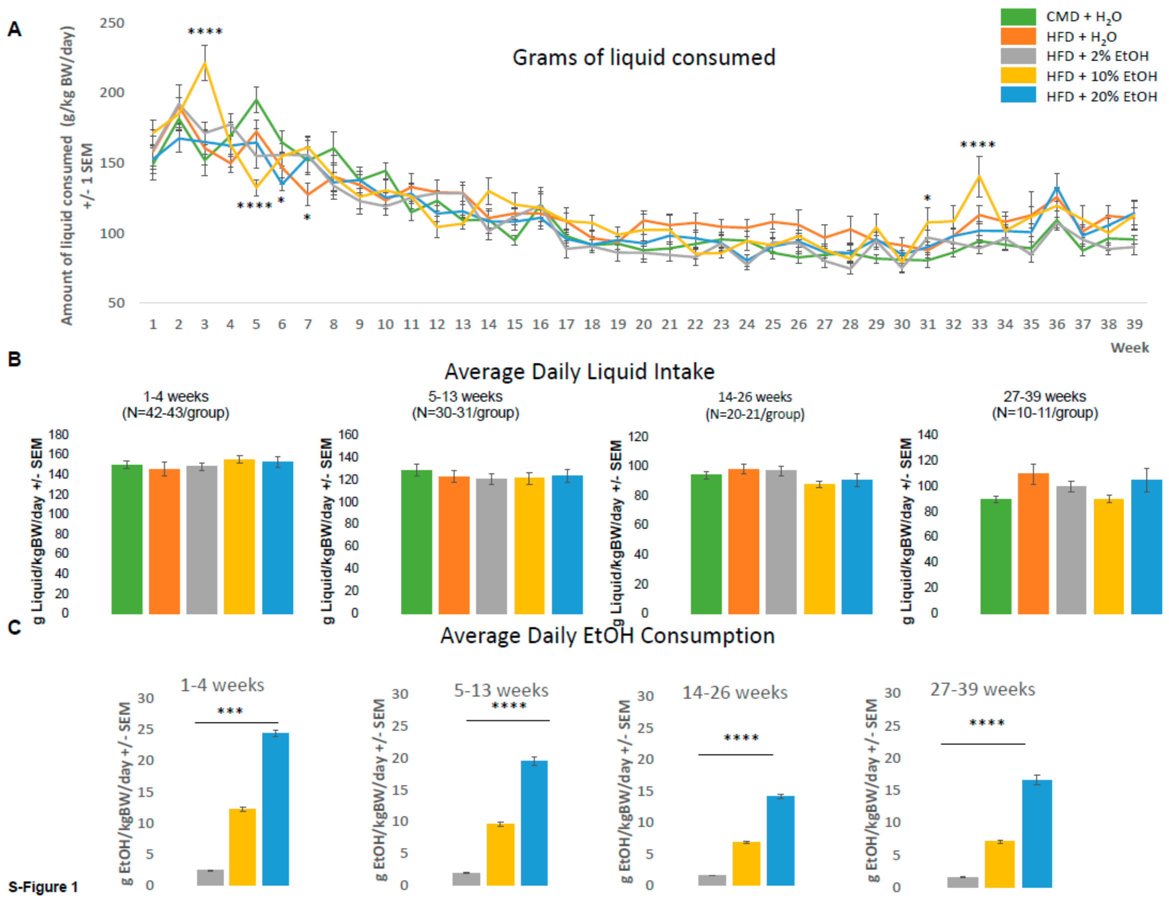

S-Figure S1. Amounts of total liquid and ethanol consumed over time. (A) Weekly average amount of liquid consumed (g/kg BW/day). Graph represents mean values +/- SEM for each week. Comparisons were made by two-way ANOVA followed by Dunnett's multiple comparisons test. Comparisons made against CMD group for a given timepoint. (B) Average daily liquid consumption (g/kg BW/day) for feeding groups during distinct time periods. Graphs represent mean values +/- SEM. (C) Average daily EtOH intake (gEtOH/kg BW/day) for HFD + 2%, 10%, or 20% E groups during distinct time periods. Graphs represent mean values +/- SEM. From weeks 1-4, N=42-43/group. From weeks 5-13, N=30-31/group. From weeks 14-26, N=20-21/group. From weeks 27-39, N=10-11/group. Comparisons were made by one-way ANOVA followed by Dunnett's multiple comparisons test. Comparisons made against CMD group for a given timepoint. Statistical significance was labeled as follows: * as $p < 0.05$; *** as $p < 0.001$; and **** as $p < 0.0001$.

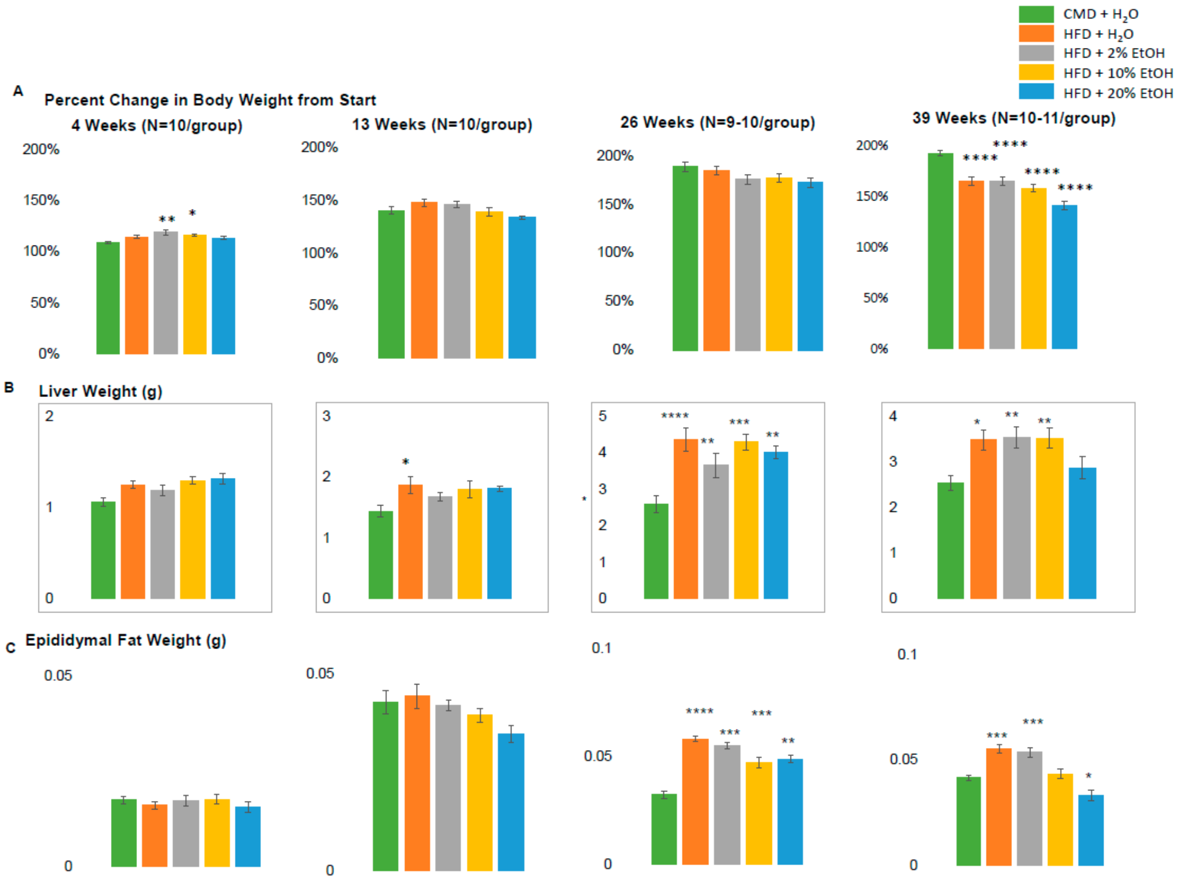

S-Figure 2

S-Figure S2. (A) Changes in BW between the beginning and end of feedings demonstrate lowest weight gains in HFD + 20% E group. (B) Changes in liver weight at all feeding groups and length, with increased liver weight in all groups. The 20% E group has less liver weight gain after 39 weeks of feeding. (C) Epididymal fat was increased in all groups with a negative association between EtOH concentration and epididymal fat weight. Graphs represent mean values +/- SEM, with N=9-12/group. Comparisons were made by one-way ANOVA followed by Dunnett's multiple comparisons test. Comparisons made against CMD group for a given timepoint. Statistical significance was labeled as follows: * as $p < 0.05$; ** as $p < 0.01$; *** as $p < 0.001$; and **** as $p < 0.0001$.

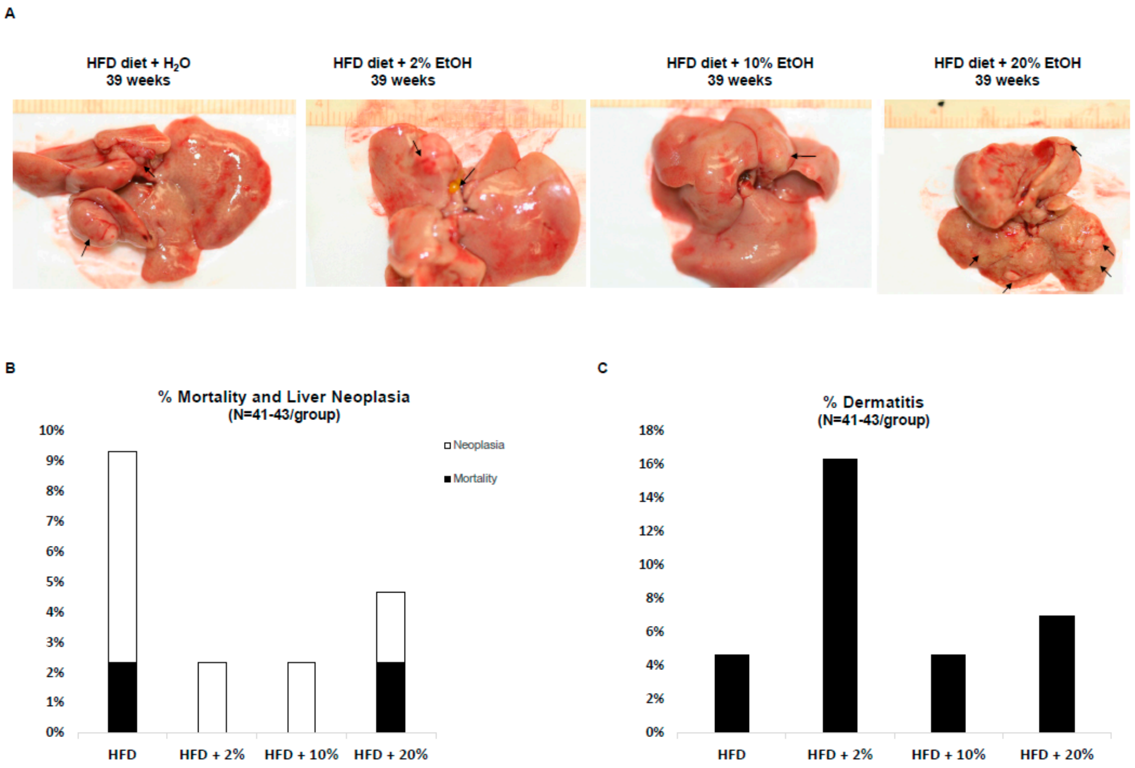

	Total	Tumor	Tumor %	Mortality	Mortality %
CMD	43	0	0%	0	0%
HFD	43	3	7%	1	2%
HFD+2%	43	2	5%	0	0%
HFD+10%	43	0	0%	0	0%
HFD+20%	43	1	2%	1	2%

S-Figure 3

S-Figure S3. Premature mortality, liver neoplasia, and dermatitis. (A) Representative neoplasm-bearing livers from the different feeding groups at 39 weeks. Arrows indicate the distinct neoplasms. (B) The graph represents the percentage of mice from each feeding group experiencing either premature death or liver neoplasia relative to the total from each group studied at all time points. (C) The graph represents the percentage of mice from each feeding group experiencing dermatitis relative to the total from each group studied at all time points. Graphs represent mean values, with N=41-43/group. Comparisons were made by one-way ANOVA.
